# New Classification and Its Value Evaluation for Atlantoaxial Dislocation

**DOI:** 10.1111/os.12734

**Published:** 2020-07-25

**Authors:** Tan Mingsheng, Gong Long, Yi Ping, Yang Feng, Tang Xiangsheng, Ma Haoning, Hao Qinying

**Affiliations:** ^1^ Department of Orthopedics, China–Japan Friendship Hospital Peking Union Medical College, Chinese Academy of Medical College Beijing 100029 China

**Keywords:** Atlantoaxial dislocation, Classification, Reliability test

## Abstract

**Objective:**

To introduce the TOI classification system (the Traction reduction type [T type], Operation reduction type [O type], and Irreducible type [I type] classification system) and to determine the interobserver and intraobserver reliability of the TOI classification system.

**Methods:**

Based on the characteristics of atlantoaxial dislocation (AAD), AAD was divided into Traction reduction type (T type), Operation reduction type (O type), and Irreducible type (I type). The analysis of interobserver and intraobserver agreements was done using kappa statistics. From July 2016 to January 2019, 213 AAD patients were retrospectively studied at four hospitals. Plain radiographs including extension and flexion views and three‐dimensional CT images were obtained. Twenty independent observers, including eight experienced spine specialists and 12 orthopaedic fellows from four different residency training hospitals, completed the survey.

**Results:**

The classification of the TOI system was based on etiology, the course of the disease, flexion–extension X‐rays, three‐dimensional CT reconstruction, and curative effects of skull traction. Flexion–extension X‐rays demonstrating a successful reduction of the dislocated atlantoaxial joint and three‐dimensional CT images showing osseous fusion of atlantoaxial facet joints and cervical traction reveal characteristics of T‐type. Furthermore, this type can be divided into two subtypes, T1 and T2, according to the etiology and course of the disease. Unsatisfactorily reduction after 1–2 weeks of strict cervical traction, no reduction shown on flexion–extension X‐rays, and no destruction or boneless fusion of atlantoaxial facet joints demonstrated in three‐dimensional CT images are characteristics of type O. Atlantoaxial facet joint showing bone fusion or failure of reduction after cervical traction or three‐dimensional CT images showing failure of surgical release are characteristics of type I. Interobserver and intraobserver reliability of the TOI classification system were moderate (κ = 0.543) and substantial (κ = 0.658), respectively. Interobserver and intraobserver reliability of the treatment choice were moderate (κ = 0.568) and substantial (κ = 0.675), respectively. There were no significant differences in the interobserver and intraobserver reliability between experienced spine specialists and fellows for all κ‐values (*P* > 0.05).

**Conclusions:**

The TOI classification system had satisfactory reliability and, therefore, can be applied clinically and used by less experienced surgeons. We believe TOI can help surgeons choose appropriate treatment strategies.

## Introduction

Atlantoaxial dislocation (AAD) is an anatomical abnormality of the atlantoaxial joint caused by trauma, degeneration, tumors, congenital malformation, pharyngeal inflammation, and surgery. Joint dysfunction and/or cord compression are present in AAD. The general incidence of AAD has not been reported yet. However, in the last two decades, the incidence of atlantoaxial fractures and dislocations caused by traffic accidents and other deceleration injuries has been on the rise, due to the high mobility of the C_1_–C_2_ joint in particular. They generally account for one‐third of all cervical spine injuries ^1^. AAD often leads to death, and the mortality rate of patients with medulla oblongata injuries is 10%–20%.^2^ In an autopsy series, 24.4% of patients whose death was attributable to traffic accidents had radiological lesions of the upper cervical spine.^3^

The treatment and classification of AAD remain controversial. In 1968, Greenberg first classified AAD into two types according to whether or not it could be reduced.^4^ In 1977, Fielding reported common atlantoaxial rotational dislocations and fixations in children.^5^ In 1991, Stauffer classified traumatic AAD into four types.^6^ In 2003, Yin Qingshui classified traumatic AAD into three types: (i) easily reversible type; (ii) reversible type with difficulty; and (iii) irreversible type.^7^ In 2004, according to reduction by skull traction, Dang Gengting divided traumatic AAD into two types: (i) reversible dislocation; and (ii) irreversible dislocation or fixed dislocation.^8^ The abovementioned classification systems have two limitations. First, the pathological state fails to correspond to its respective type. For example, as for irreversible dislocation by skull traction, both pathological states, whether the atlantoaxial facet joint is destroyed and fused in dislocation state or that is not done but in dislocation state due to ligament, muscle contracture, and/or scar fixation. Second, these classification systems do not clearly describe AAD caused by acute injury of the atlantoaxial region, dislocation, and pharyngeal inflammation. Therefore, the current classification systems for AAD do not contribute to these patients’ decision‐making regarding treatment.

The traditional surgical treatment for atlantoaxial dislocation was mainly occipitocervical multi‐segment fixation and fusion *via* a posterior approach. It was difficult for an old dislocation to achieve adequate decompression, reduction, and fixed fusion. In recent years, the progress in clinical techniques has helped solve clinical problems associated with atlantoaxial dislocation, such as those relating to surgical release, decompression, reduction, fusion by short‐segment fixation, and bone grafting. However, due to the different causes, methods, treatments, and prognosis of the dislocation, and the significance of the anatomical structure of this position, the classification remains controversial.

In 2007, our teams proposed the surgical classification of atlantoaxial dislocation (TOI, Traction reduction type, T type/Operation reduction type, O type/Irreducible type, I type), based on the above research.^9^ The purpose of this study is: (i) to introduce the classification of TOI for AAD and to determine the interobserver and intraobserver reliability of the classification system of TOI; (ii) to assess whether the classification of TOI has good applicability and reliability in the treatment of AAD; and (iii) to discuss the advantages of the TOI classification system. We demonstrate that this classification system for AAD has good reliability and will help surgeons to create treatments plan for patients with AAD.

## Materials and Methods

### *Patients*

Ethical approval for this study was obtained by the Ethics Committee of the Department of Orthopedic Surgery in our hospital on 1 July 2016 with written informed consent. This study was conducted in accordance with the Helsinki declaration. We performed a prospective study in our hospital to enroll 213 consecutive patients with AAD from July 2016 to January 2019 at four hospitals (Table 1).

**TABLE 1:** Demographic characteristics and baseline information

Variables	Data
Age (years)	47.6 (24–62)
Gender (female/male)	165/48 (77.5%/22.5%)
Causes (the number/percentage)	
Trauma	130 (61.0%)
Occipitocervical malformation	33 (15.5%)
Odontoid process nonunion	29 (13.6%)
Iatrogenic instability	7 (3.3%)
Atlantoaxial tumors	6 (2.8%)
Pharyngitis	3 (1.4%)
Atlantoaxial tuberculosis	2 (0.9%)
Ankylosing spondylitis	2 (0.9%)
Rheumatoid arthritis	1 (0.5%)
Neurological status	
Before treatments	
Normal	96 (45.1%)
Mild and moderate	71 (33.3%)
Severe and extremely severe	46 (21.6%)
After treatments	
Normal	165 (77.5%)
Mild and moderate	30 (14.1%)
Severe and extremely severe	18 (8.4%)

Inclusion criteria were: (i) clinical presentation and imaging features consistent with atlantoaxial dislocation or instability; and (ii) the diagnostic criteria of imaging were atlas‐dens interval (ADI) >5 mm or space available for the cord (SAC) < 13 mm.

Exclusion criteria were (i) patients with severe cardiopulmonary dysfunction and at high risk for undergoing surgery; (ii) severe disorder of the spinal cord (patients meeting Symon and Lavender’s criteria were classified as extremely severe); and (iii) severe occipitocervical deformities, skull base depression, and normal ADI and SAC.

No patients included in this study suffered further neurological deficits during the imaging period. Japanese Orthopedic Association (JOA) scoring^10^ and Symon & Lavender clinical standards^11^ were used to evaluate the neurological function and clinical outcome. The Symon and Lavender criteria classified patients’ functional states into four grades: (i) mild, only slight dysfunction, and normal work; (ii) moderate, obvious dysfunction, and partial work; and (iii) severe, unable to work and walk only indoors; and (iv) extremely severe, unable to get out of bed, stand or walk. Demographic data and baseline information are detailed in Table 1. Patients were followed up for 1 to 13 years (on average, 6.8 years), and their neurological function was evaluated before treatment and at the last follow‐up.

### *Images*

Plain radiographs including extension and flexion views and three‐dimensional CT images were obtained. The patients had extension and flexion X‐rays only following evaluation for safety by experienced surgeons specialized in the upper spine. If a patient suffered severe trauma and obvious signs of instability, the patient was not given the dynamic X‐rays.

### *Investigators and Survey*

Twenty independent observers, including eight experienced spine specialists and 12 orthopaedic fellows from four different residency training hospitals, completed the survey. The spine specialists’ 14.2 years of experience on average (range, 10 to 21 years). After fully understanding the classification system, they independently classified AAD in 213 patients. A paper questionnaire was adopted. Images from 213 patients and information, including their age, sex, case history, and condition, was randomly presented to the observers. For each case, three questions were asked: (i) “What is the type of this AAD according to the new classification system (TOI)?”; (ii) “What would be your treatment of choice for this AAD, conservative or operative?”; and (iii) “If operative, what would be your surgical plan for this AAD?” To evaluate intraobserver reliability, the observers repeated the same procedure with randomization 8 weeks after the first round of assessment.

### *Statistical Methods*

Interobserver reliability was evaluated to determine the reliability of the opinions of different observers for each case. By contrast, intraobserver reliability was evaluated to determine the reliability of individual observers by comparing the first and second surveys for each case.

Interobserver and intraobserver reliability were evaluated by calculating the correlation coefficient, as described by Fleiss.^7^ The *κ*‐values were interpreted according to Landis and Koch criteria.^8^ The *κ*‐values were used to analyze interobserver and intraobserver agreement. The result was interpreted according to the following criteria: <0 corresponded to no agreement, 0.00 to 0.02 corresponded to slight agreement, 0.21 to 0.40 corresponded to fair agreement, 0.41 to 0.60 corresponded to moderate agreement, 0.61 to 0.80 corresponded to substantial agreement, and 0.81 to 1.0 corresponded to almost perfect agreement. The paired *t*‐test was used to determine the statistical significance of differences between mean values. The statistical significance and the power analysis were set at *P*‐value ≤0.05 and 0.8. All analyses were performed using SPSS version 22 (SPSS; Chicago, IL, USA).

## Results

### *Proposed Classification System*

**Fig. 1:**
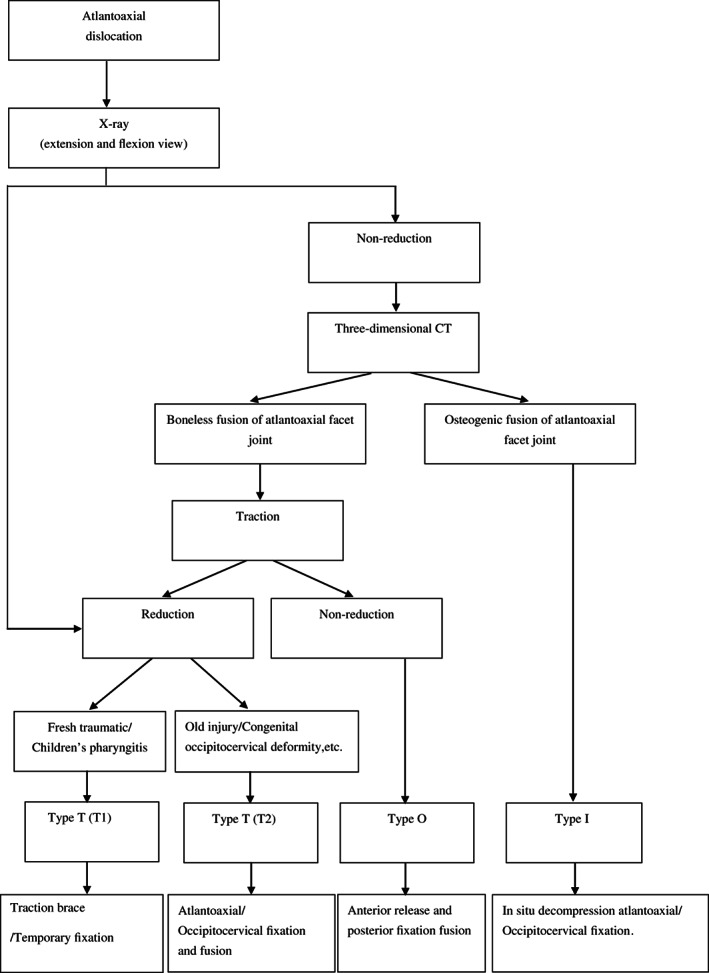
Treatment diagram according to the TOI classification system for atlantoaxial dislocation.

The classification of the TOI system was based on etiology, course of the disease, flexion–extension X‐ray, three‐dimensional CT reconstruction, and curative effects of skull traction (see Fig. 1).

#### *Traction Reduction Type (T‐type)*

Flexion–extension X‐ray showing a successful reduction of the dislocated atlantoaxial joint and three‐dimensional CT images without osseous fusion of atlantoaxial facet joints and cervical traction reflect T‐type characteristics. Furthermore, this type can be divided into two subtypes, T1 and T2, according to the etiology and course of the disease. Of note, whether a patient with AAD can be reducible or not depends on a combination of flexion–extension X‐rays and reducibility intraoperatively under anesthesia on the operating table and not just X‐rays.

T1: Atlantoaxial dislocation resulting from fresh atlantoaxial trauma of less than 3 weeks and pharyngeal inflammation of children. Atlantoaxial stability and function can be obtained after reduction and fixation.

T2: Atlantoaxial dislocation including diseases and trauma (congenital occipitocervical malformation, atlanto‐occipital fusion, nonunion of the odontoid process, old upper cervical spine injury >3 weeks, Jefferson fracture with a separation of lateral mass ≥ 6.9 mm, severe atlanto‐occipital joint injury, comminuted fracture of the lateral mass of atlas, transverse ligament rupture (ADI ≥5 mm), type II odontoid fracture, tuberculosis, tumors, rheumatoid arthritis, and degenerative instability). Its stability must be obtained by reduction, internal fixation, and bone grafting.

#### *Operational Reduction Type (O‐type)*

Satisfactorily reduction after 1–2 weeks of strict cervical traction, no reduction evident on flexion–extension X‐ray, and no destruction or boneless fusion of atlantoaxial facet joints in three‐dimensional CT images were characteristics of type O, such as old atlantoaxial fracture and dislocation, failure of upper cervical spine surgery, and congenital occipitocervical deformity.

#### *Irreducible Type (I‐type)*

Atlantoaxial facet joint showing bone fusion, failure of reduction after cervical traction, and failure of surgical release in three‐dimensional CT are characteristics of type I, such as serious deformity or dislocation with bony destruction and fusion resulting from old fractures of the upper cervical spine, rheumatoid arthritis, ankylosing spondylitis, and iatrogenic instability.

### *Interobserver and Intraobserver Reliability of the TOI Classification System*

Interobserver and intraobserver reliability of the TOI classification system was moderate (*κ* = 0.543) and substantial (*κ* = 0.658), respectively. There were no significant differences in the interobserver and intraobserver reliability between experienced spine specialists and fellows (*κ* = 0.587 *vs* 0.562, *κ* = 0.645 *vs* 0.671, respectively) (*P* = 0.135, *P* = 0.125) (Table 2).

**TABLE 2:** Interobserver and intraobserver reliability of TOI classification system for atlantoaxial dislocation

	Specialist group	Fellow group	*P*‐value	*κ‐*value
Interobserver reliability	0.587	0.562	0.135	0.543
First round	0.545	0.578	‐	0.540
Second round	0.629	0.546	‐	0.546
Intraobserver reliability	0.645	0.671	0.125	0.658

### *Interobserver and Intraobserver Reliability of Treatment Choice*

Interobserver and intraobserver reliability of the treatment choice was moderate (*κ* = 0.568) and substantial (*κ* = 0.675), respectively. There were no significant differences in the level of interobserver and intraobserver reliability between experienced spine specialists and orthopaedic fellows (*κ* = 0.624 *vs* 0.543, *κ* = 0.727 *vs* 0.659, respectively) (*P* = 0.165, *P* = 0.158) (Table 3).

**TABLE 3:** Interobserver and intraobserver reliability of treatment choice for atlantoaxial dislocation

	Specialist group	Fellow group	*P*‐value	*κ‐*value
Interobserver reliability	0.522	0.612	0.165	0.568
First round	0.540	0.635	‐	0.575
Second round	0.504	0.589	‐	0.532
Intraobserver reliability	0.635	0.705	0.158	0.675

## Discussion

The current study demonstrated moderate interobserver and substantial intraobserver reliability of the TOI classification system for AAD and the corresponding treatment choice, which is of significance for the classification systems for AAD and further benefit on its treatment. The proposed classification for AAD followed the standardized process for creating a new classification system.^7^, ^8^

An ideal classification system is reproducible, with a high level of interobserver and intraobserver reliability. It should be able to indicate the appropriate treatment path and to predict the outcome.^12^ Several classification systems for AAD, such as Greenberg’s, Fielding’s, and Dang Gengting’s classifications, have been proposed.^4^, ^5^, ^6^, ^7^, ^8^ However, these classification systems are not specific for AAD and do not consider the entire AAD.

Greenberg’s classification for AAD, which is based on whether it could be reduced or not, has been widely used since 1968.^4^ In 1977, Fielding summarized common atlantoaxial rotational dislocations and fixations in children.^5^ In 1991, Stauffer introduced a more detailed classification system by expanding the original Greenberg classification system.^6^ This classification system is most commonly used for the diagnosis and treatment of AAD. Many surgeons rely on Stauffer classification systems to direct decision‐making for treatment. However, there are AAD that are unclassifiable using these systems, such as those caused by acute injury of the atlantoaxial region and pharyngeal inflammation, resulting in confusion about appropriate treatment. In addition, there are few studies that test of the reliability of these classification systems.^1^, ^6^ The confusion among various types of atlantoaxial dislocations, the inconsistency of criteria, and the inaccuracy of evaluation directly lead to undertreatment or overtreatment, problems that burden the individual and society. For these reasons, in 2007, we devised a new classification system for AAD taking into account acute injuries of the atlantoaxial region and pharyngeal inflammation as well as different pathological states to provide better information for diagnosis and treatment. Our TOI system classifies AAD into three types, with two subtypes for T‐type, based on etiology, course of the disease, flexion–extension X‐ray, three‐dimensional CT reconstruction, and curative effects of skull traction. For T‐Type 1, atlantoaxial stability and function can be obtained after reduction and fixation. For T‐Type 2, atlantoaxial stability must be obtained by reduction, internal fixation, and bone grafting. For O‐type, posterior decompression, reduction, internal fixation, bone graft, and fusion were performed after oropharyngeal release. In addition, neck circumference or neck or head–neck‐chest plaster was used to immobilize the neck for 10–12 weeks. For I type, treatments including *in situ* decompression, internal fixation, and bone graft fusion were adopted. For patients whose C_1_–C_2_ was already fused, fusion is necessary, because the biochemical characteristic in the tissue of the fusion is fragile and unstable, especially after the decompression, and patients are typically elderly with decreased bone mass and osteoporosis. A cervicothoracic brace or head–neck–chest plaster strictly immobilized the neck for 10 to 12 weeks postoperatively. Of note, for O type and I type, decompression is necessary.

For those classification systems mentioned above, their reliability was not adequately evaluated. In the current study, the interobserver and intraobserver reliability of our TOI classification system were moderate and substantial (κ = 0.543 and κ = 0.658), respectively. Interobserver and intraobserver reliability for treatment choice were moderate and substantial (κ = 0.568 and κ = 0.675), respectively. We detected no significant differences in the interobserver and intraobserver reliability between experienced spine specialists and fellows. Our results suggested that the TOI classification system had satisfactory reliability. Therefore, it can be applied clinically and can be undertaken by less experienced surgeons.

At present, there are no studies that introduce a treatment algorithm for AAD. With the development of surgical techniques, traction is no longer the only method for the reduction of atlantoaxial dislocation. Operative release and the pedicle screw technique have been able to achieve reduction in the dislocated atlantoaxial joint when traction reduction has been unsuccessful.^13^, ^14^, ^15^ Therefore, considering operative release, the technique of three‐dimensional CT and traumatic factors, we should distinguish atlantoaxial dislocations that can be reduced surgically from the others. In the present study, our TOI classification system is able to do that. It also further generalizes the existing classification methods of AAD. It has far‐reaching clinical significance. There are several unclassifiable AAD that could not be categorized using previous classifications, such as those caused by acute injury of the atlantoaxial region and pharyngeal inflammation, resulting in confusion about appropriate treatment, that can be classified using the presently proposed methods. This directly helps control undertreatment or overtreatment and reduces individual and social burdens. Therefore, the classification of AAD is more clinically practical, clearly defined, and instructive for the treatment of this disease in comparison to previous classification systems.

Any classification system should help the practitioner achieve two purposes, including the creation of a common method for individuals who choose appropriate treatments, thereby promoting efficient and reliable communication, and assistance in clinical decision‐making related to the need for operative *versus* non‐operative care, the surgical approach, and outcome prediction.^16^ The TOI classification system achieves these two aims, and, therefore, we believe that the TOI classification system will be helpful for constructing standard therapeutic strategies for AAD.

There are several limitations in this study. First, this was merely a study evaluating the reliability of the classification system, and not a systematic study. As specific types of AAD could be controversial among even experienced spine specialists, the TOI system’s sensitivity and specificity could not be assessed at present. Admittedly, external validation is important. However, the present study primarily introduces the classification of TOI for AAD and determines the interobserver and intraobserver reliability of the classification system of TOI. We would provide an external validation cohort to test it in further research. Second, the TOI classification system is not suitable for patients with severe congenital occipitocervical malformation, basilar invagination, obvious compression of the ventral brainstem, and spinal cord without abnormal ADI and SAC. Third, the surgical treatment guided by TOI classification has these following situations, including the same type which can have different treatment options and the same surgical method, which can be used for different types of AAD. Therefore, satisfactory results require a comprehensive analysis of each case’s characteristics, surgical indications, rational treatment options, merits and demerits of the surgical method, medical team’s skill in the proposed surgical procedures according to the ﬂow chart of the TOI classiﬁcation system. Fourth, there existed recall bias when applying a survey with the same images repeated. However, we randomly presented the images of each patient to the observers during each survey with an interval of 8 weeks to minimize this effect. These limitations would open the door for future studies.

### *Conclusion*

The treatment and classification of AAD remain controversial. Our study revealed moderate interobserver reliability and substantial intraobserver reliability of our TOI classification system and treatment choice. Because the TOI classification system has satisfactory reliability, it can be applied clinically and used by less experienced surgeons. Therefore, the TOI classification system can help surgeons choose appropriate treatment strategies.
